# Induced pluripotent stem cells: applications in regenerative medicine, disease modeling, and drug discovery

**DOI:** 10.3389/fcell.2015.00002

**Published:** 2015-02-02

**Authors:** Vimal K. Singh, Manisha Kalsan, Neeraj Kumar, Abhishek Saini, Ramesh Chandra

**Affiliations:** ^1^INSPIRE Faculty, Stem Cell Research Laboratory, Department of Biotechnology, Delhi Technological UniversityDelhi, India; ^2^Stem Cell Research Laboratory, Department of Biotechnology, Delhi Technological UniversityDelhi, India; ^3^B. R. Ambedkar Centre for Biomedical Research, University of DelhiDelhi, India

**Keywords:** iPSC, reprogramming, pluripotency, differentiation, disease modeling, drug discovery, gene therapy

## Abstract

Recent progresses in the field of Induced Pluripotent Stem Cells (iPSCs) have opened up many gateways for the research in therapeutics. iPSCs are the cells which are reprogrammed from somatic cells using different transcription factors. iPSCs possess unique properties of self renewal and differentiation to many types of cell lineage. Hence could replace the use of embryonic stem cells (ESC), and may overcome the various ethical issues regarding the use of embryos in research and clinics. Overwhelming responses prompted worldwide by a large number of researchers about the use of iPSCs evoked a large number of peple to establish more authentic methods for iPSC generation. This would require understanding the underlying mechanism in a detailed manner. There have been a large number of reports showing potential role of different molecules as putative regulators of iPSC generating methods. The molecular mechanisms that play role in reprogramming to generate iPSCs from different types of somatic cell sources involves a plethora of molecules including miRNAs, DNA modifying agents (viz. DNA methyl transferases), NANOG, etc. While promising a number of important roles in various clinical/research studies, iPSCs could also be of great use in studying molecular mechanism of many diseases. There are various diseases that have been modeled by uing iPSCs for better understanding of their etiology which maybe further utilized for developing putative treatments for these diseases. In addition, iPSCs are used for the production of patient-specific cells which can be transplanted to the site of injury or the site of tissue degeneration due to various disease conditions. The use of iPSCs may eliminate the chances of immune rejection as patient specific cells may be used for transplantation in various engraftment processes. Moreover, iPSC technology has been employed in various diseases for disease modeling and gene therapy. The technique offers benefits over other similar techniques such as animal models. Many toxic compounds (different chemical compounds, pharmaceutical drugs, other hazardous chemicals, or environmental conditions) which are encountered by humans and newly designed drugs may be evaluated for toxicity and effects by using iPSCs. Thus, the applications of iPSCs in regenerative medicine, disease modeling, and drug discovery are enormous and should be explored in a more comprehensive manner.

## Introduction

**Figure 1:**
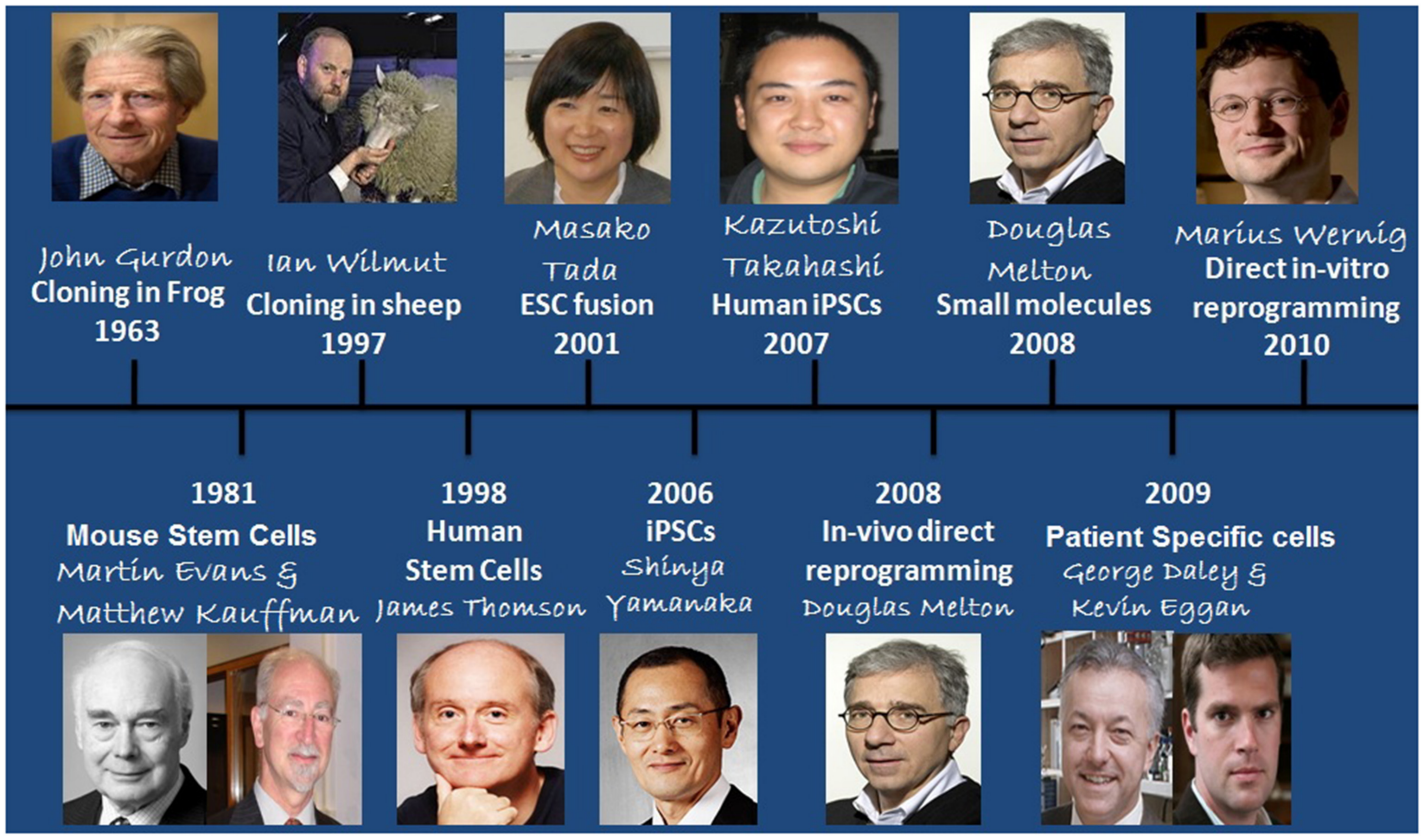
**Historical timeline showing events that led to the development of iPSCs and the recent advances that have occurred in the field**.

Discovery of self-renewal by any living cell was one of the major breakthrough reported by Till and McCulloch (1961) who while subjecting the mice with lethal doses of radiation followed by injection of bone marrow cells found that these cells formed clumps due to cells cloned from them which was the main reason of survival of the mice (Till and McCulloch, 1961). Later studies defined their potential for differentiation into different cell types and self-renewal without senescence, and termed as Stem Cells. Stem cells can be defined on the basis of their origin and potency into Adult Stem Cells and Embryonic Stem Cells (ESC), similarly, considering their potency as the base of classification, stem cells can be classified into unipotent, multipotent, oligopotent, pluripotent, and totipotent (Lan et al., 2012). Totipotent are those which can differentiate into embryonic as well as extra-embryonic tissues such as the placenta. Pluripotent stem cells are those which can differentiate into other cells of the adult body. This property exists for only a specific time period of pre-implantation development in the cells forming Inner Cell Mass (ICM). As the cells differentiate into other cell lineages, their self renewing potential decreases due to various epigenetic changes which leads to the loss of pluripotency. Further research conducted on human stem cells (HSCs) made burgeoning use of human ESCs for which embryo needed to be isolated on regular basis that evoked several ethical issues among socio-research communities. There have been efforts by different research groups to bypass these ethical/technical problems. Subsequent studies demonstrated many advancements in the related techniques such as cloning in frog (Gurdon, 1962), generation of mouse Stem Cells (SCs) (Evans and Kaufman, 1981), cloning in sheep (Wilmut et al., 1997), generation of HSCs (Thomson et al., 1998), and development of Embryonic stem cell fusion technique (Tada et al., 2001). These findings contributed greatly to the development of cells that could eliminate ethical issues (Figure 1). However, the major breakthrough came in 2006 when Takahashi and Yamanaka introduced the concept of induced pluripotent stem cells (iPSCs) by generating stem cells that were having properties relating to ESCs. iPSCs were generated by using a combination of 4 reprogramming factors, including Oct4 (Octamer binding transcription factor-4), Sox2 (Sex determining region Y)-box 2, Klf4 (Kruppel Like Factor-4), and c-Myc and were demonstrated both self-renewing and differentiating like ESCs, and thus, could be used as an alternative for hESCs in various clinics/research. Since then, a number of different reprogramming factors/methods have been established. iPSCs generation may employ combination of different reprogramming factors, viz., a cocktail of various reprogramming factors, direct use of proteins, miRNA, peptide etc. Factors (LIN28 + Nanog, Esrrb, Pax5 shRNA, C/EBPa, p53 siRNA, UTF1, DNMT shRNA, Wnt3a, SV40 LT(T), hTERT) or chemicals (BIX-01294, BayK8644, RG108, AZA, dexamethasone, VPA, TSA, SAHA, PD025901 + CHIR99021(2i), A-83-01) have been found which are able to replace one or the other reprogramming factors from basal reprogramming factors or to enhance the efficiency of reprogramming (Table 1) (Yu et al., 2007; Dimos et al., 2008; Hanna et al., 2008; Huangfu et al., 2008a,b; Mali et al., 2008; Marson et al., 2008; Mikkelsen et al., 2008; Park et al., 2008a; Shi et al., 2008a,b; Silva et al., 2008; Zhao et al., 2008; Zhou et al., 2008; Feng et al., 2009; Li et al., 2009; Fong et al., 2011).

**Table 1:** Many factors and chemicals are able to replace the basal transcriptions factors used for reprogramming (O- Oct4; S- Sox2; K- Klf4; M- c-Myc; M*- N-Myc).

**Factor/chemical**	**Function**	**Able to replace**	**References**
Nanog	ESC-specific transcription factor	Together with Lin28, able of replacing K and M	Yu et al., 2007
Lin28	ESC-specific RNA-binding protein	Together with Nanog, able of replacing K and M	Yu et al., 2007
Esrrb	Orphan nuclear receptor	K	Feng et al., 2009
SV40 LT (T)	SV40 large T antigen used for cell transformation	K; M* and Lin28, Nanog	Mali et al., 2008
BIX-01294	Inhibitor of G9a histone methyltransferase	S, O	Shi et al., 2008a,b
VPA	Inhibitor of histone deacetylase	K and M	Huangfu et al., 2008a

Similarly, there have been significant advancements in methods/techniques employed to deliver these factors such as Nuclear Transfer, Transcription Factors (Oct4, Sox2, Klf4 and c-Myc) and different reprogramming technologies, including Integration viral vectors and Integration free vectors (Plasmid DNA and transferase, Viral vectors, and Excision of integrated transgenes) (Hochedlinger and Jaenisch, 2002; Takahashi and Yamanaka, 2006a; Takahashi et al., 2006b; Yu et al., 2007; Stadtfeld et al., 2008a,b,a,b; Fusaki et al., 2009; Gonzalez et al., 2009; Yusa et al., 2009) (Table 2). The various sources, delivery methods and their cognate approaches with different combinations of transcription factors differ significantly in their efficiency (Tables 2, 3).

**Table 2:** **Different delivery methods for transfer of different combinations of transcription factors have different efficiencies of reprogramming (O- Oct4; S- Sox2; K- Klf4; M- c-Myc)**.

	**Reprogramming**	**Factors**	**Cell type**	**Efficiency %**	**References**
Integrating methods	Retroviral transduction	OSKM	Mouse fibroblast	0.001–1	Takahashi and Yamanaka, 2006a
		OSK + VPA	Neonatal	1	Huangfu et al., 2008a
	Lentiviral	OSKM	Human fibroblast	0.1–1	Yu et al., 2007
		OK + parnate + CHIR99021	Neonatal	0.02	Li et al., 2009
	Inducible lentiviral	OSKM	Human fibroblast	0.1–2	Maherali et al., 2008
Non-integrating methods	Sendai virus	OSKM	Human fibroblast	~0.1	Fusaki et al., 2009
	Adeno viral transduction	OSKM	Mouse fibroblast	~0.001	Stadtfeld et al., 2008a
	Plasmid DNA transfer	OSK	Fibroblast	0.00	Okita et al., 2008
	lox p lentivirus	OSKM	Fibroblast	0.1–1	Somers et al., 2010
	PiggyBAC	OSKM	Fibroblast	0.01	Woltjen et al., 2009
	Polyarginine tagged polypeptide	OSKM	Neonatal fibroblast	0.00	Kim et al., 2009b
	RNA modified synthetic mRNA	OSKM	Human fibroblast	4.40	Warren et al., 2010

**Table 3:** **Different cell sources and different combinations of reprogramming factors have been used by different groups for reprogramming to iPSCs (O- Oct4; S- Sox2; K- Klf4; M- c-Myc; N- Nanog, L- Lin28)**.

**Type of cells**	**Reprogramming factors**	**References**
Fibroblast	OSKM	Takahashi and Yamanaka, 2006a
	OSLN	Yu et al., 2007
Keratinocytes	OSKM	Aasen et al., 2008
Cord blood endothelial cells	OSLN	Haase et al., 2009
Cord blood stem cells	OSKM	Ye et al., 2009
Neural stem cells	O	Kim et al., 2009a
Melanocytes	OSKM	Utikal et al., 2009
Amniotic cells	OSKM	Li et al., 2009
Adipose derived stem cells	OSKM	Sugii et al., 2010
Hepatocytes	OSKM	Liu et al., 2010
Circulating T cells	OSKM	Seki et al., 2010
Astrocytes	OSKM	Ruiz et al., 2010
Peripheral blood	OSKM	Kunisato et al., 2011
Kidney mesangial cells	OSKM	Song et al., 2011
Urine cells	OS	Zhou et al., 2012

Since their discovery, iPSCs have been used in many research and clinical studies including *disease modeling, regenerative medicine, and drug discovery/drug cytotoxicity studies*. For disease modeling, somatic cells possessing the characteristics of diseased cells from patient are used for the generation of iPSCs and are further utilized for studying the diseases. Similarly, iPSCs are widely utilized for the regeneration of tissue-specific cells for the transplantation to patients of various injuries or degenerative diseases. Studies like drug discovery which implies screening of small molecules, testing of toxicity for assessment of safety, have successfully exploited iPSCs based technique. A large number of reports have accumulated indicating a need for discussing various apects of these techniques and their future perspectives in clinical/research studies. This article focuses the importance of these cells for therapeutics and research benefits in different biological sciences.

## Regulatory factors of iPSCs generation: an overview

### Generation of iPSCs

**Figure 2:**
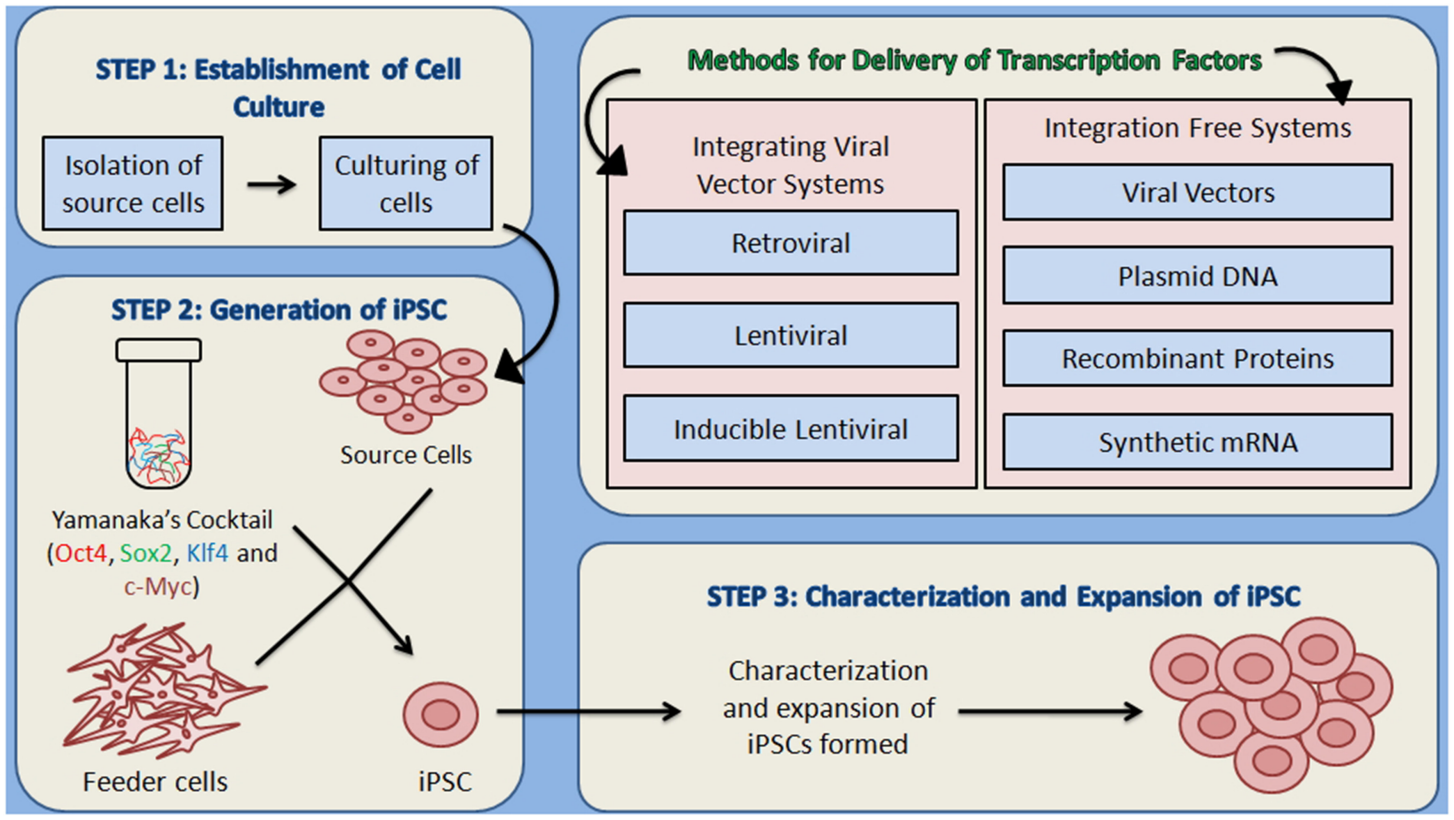
**An overview of the methodology for the generation of iPSCs**. (1) Establishment of culture: the source cells are cultured for further use as host cells for the delivery of reprogramming proteins. (2) The cultured source cells are then transfected with the four factors from Yamanaka's cocktail and incubated on feeder layers that provide the nourishment to host cells and are responsible for the formation of extra cellular matrix, under suitable conditions of media. Two types of methods for the delivery of reprogramming factors into the somatic cells can be used- Integrating viral vector systems and Non-Integrating methods. (3) After the formation of iPSCs, they are characterized by different morphological and physicochemical analyses, which is followed by the expansion of iPSCs.

**Figure 3:**
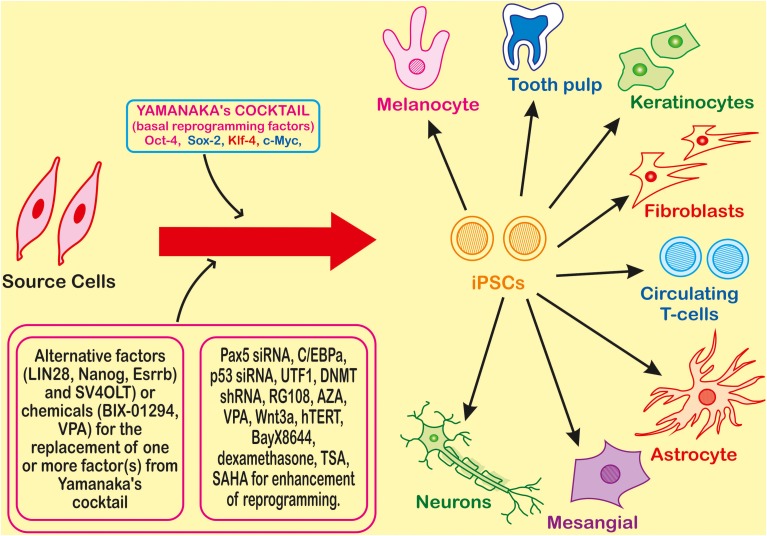
**Introduction of the four transcription factors (Oct-4, Sox-2, Klf-4, and c-Myc) leads to reprogramming of a somatic cell to an Induced Pluripotent Stem Cell (iPSC) which can further differentiate into different types of cells**. Many factors or chemicals are able to replace one of the factors from the basic four factors required for reprogramming, and for the enhancement many other small molecule chemicals or factor are also used.

Theoretically, iPSCs can be generated by using any somatic cell by employing appropriate reprogramming factors and most convenient method for their introduction to somatic cells. iPSC generation is reported by using cells from different sources such as fibroblasts, cord blood, peripheral blood (Table 1). There are large numbers of reports showing the whole process in a detailed manner that can be summarized in three major steps—(i) establishment of initial cell culture, (ii) induction of iPSCs and, (iii) characterization and expansion of iPSCs (Figure 2). Firstly, the source cells are isolated and cultured. The reprogramming factors are then introduced into those cultured cells. There are two types of methods for the introduction of reprogramming factors- Integrating Systems (retroviral, lentiviral, inducible lentiviral) and Non-Integrating Methods [non-intergrating viral vectors (sendai virus, adeno virus), plasmid DNA transfer, transgenes, recombinant proteins, synthetic mRNA] (Table 2). These transfected cells are incubated on feeder layers (a number of cells have been demonstrated as feeder cells, example., fibroblasts, keratinocytes) under appropriate media conditions and after the expression of these reprogramming factors, generation of iPSCs occurs. The cultured iPSC colonies can be characterized by different morphological and physiochemical methods. The morphological examination of iPSCs can be done on the basis of round shape, large nucleolus, and scant cytoplasm of iPSCs as similar to ESCs (Thomson et al., 1998). Reprogrammed colonies are tightly packed, sharp edged, flat, mitotically very active due to the possession of property of self renewal. Though it is hard to define iPSCs solely on the basis of their morphology, these characteristics give a quick idea about their states. Further, iPSCs may be defined on the basis of expression of different cell surface proteins (SSEA-4, alkaline phosphatase) and transcription factors Oct4, Sox2, Nanog which can also be used for the characterization (Adewumi et al., 2007). Figure 3 describes the necessary requirements (Yamanaka's cocktail, alternative factors or chemicals for the replacement of one or more basal reprogramming factors enhancement of efficiency of generation) for the generation of iPSCs.

### Different factors that play role in the generation of iPSCs

As discussed above, the generation of iPSCs from somatic cells requires the introduction of reprogramming factors into the somatic cells. This can be done by two types of methods—Integrating Viral Vector Systems and Non-Integrating Systems. Integrating methods are those in which the viral vector gets integrated into the host cell genome. The use of retrovirus and lentivirus comes under this category (Takahashi et al., 2006b; Brambrink et al., 2008; Stadtfeld et al., 2008a,b). This method has a high efficiency but it poses the risks of cancer formation and hence, different approaches have also been worked on. Non-Integrating methods are those in which no integration in host cell genome occurs. Different approaches such as viral vectors (Adeno virus and Sendai virus), Plasmid DNA, and the use of RNA and proteins come under this category. Adeno virus (dsDNA) and Sendai virus (RNA) are non-integrating viral vectors which have been used in iPSC generation (Takahashi et al., 2006b; Stadtfeld et al., 2008a; Fusaki et al., 2009). Plasmids such as oriP/EBNA1 (derived from Epstein-bar virus), containing reprogramming factors has been used for reprogramming, but the efficiency was found to be low (Yu et al., 2009). Direct delivery of reprogramming proteins has also been carried out by fusing them with a cell penetrating peptide (Kim et al., 2009b). Yashioka et al. used a different approach using a single self replicating RNA replicon which expressed high levels of Yamanaka's factors for transfection into fibroblasts to be reprogrammed into iPSCs and found the iPSCs to be having all properties of pluripotent stem cells (Yoshioka et al., 2013).

Once the reprogramming factors are delivered to the somatic cells, they come into play for transforming those somatic cells into iPSCs. Not only they have their individual roles, but they interact with each other complementarily.

## Applications of iPSCs

**Figure 4:**
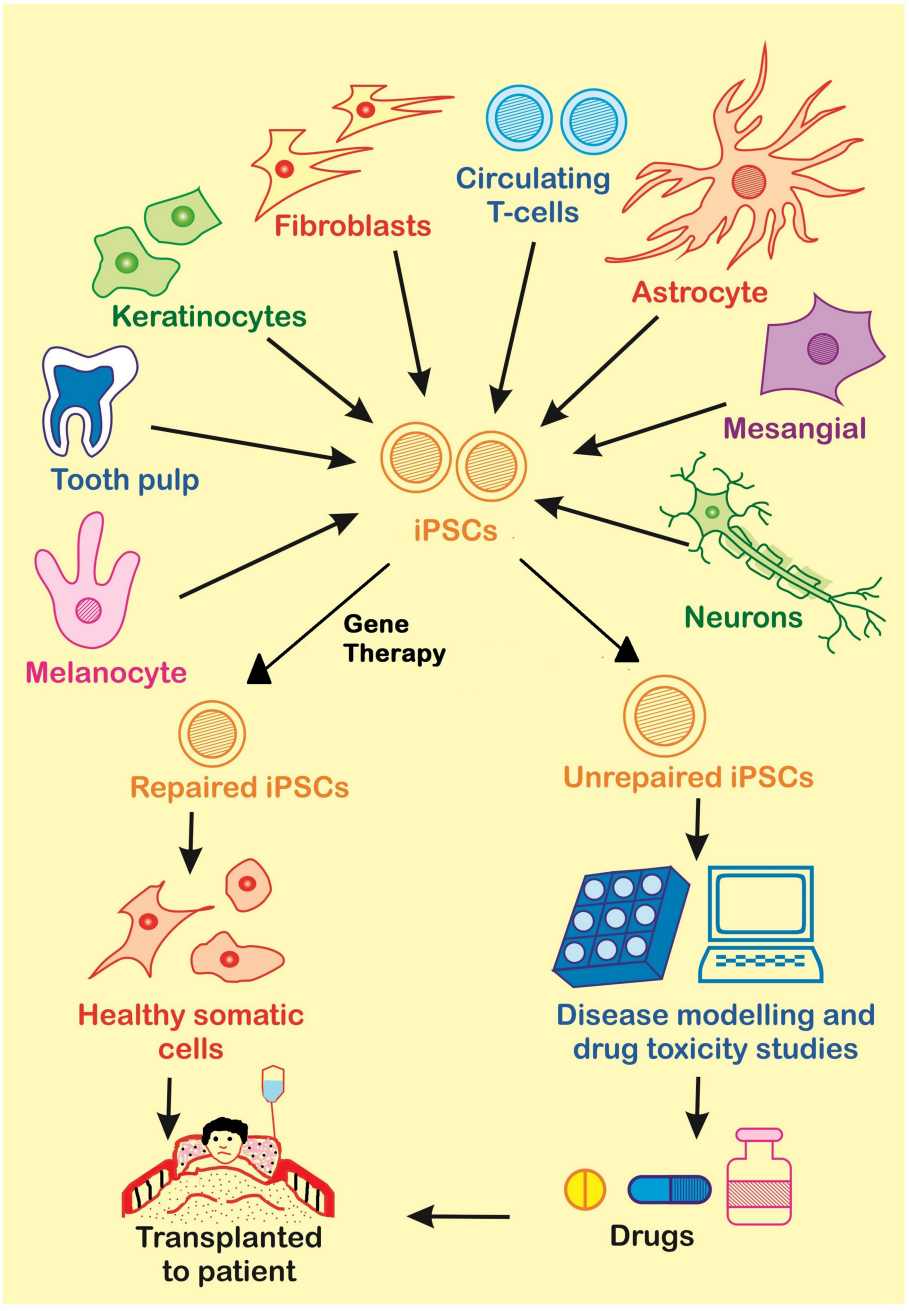
**There are many applications of iPSCs in the fields of gene therapy, disease modeling and drug discovery**. Somatic cells from the patient are used for the generation of diseased iPSCs. These diseased iPSCs may be repaired by Gene Therapy and further used for the generation of healthy somatic cells to be transplanted to the patient, or they may be used to produce unrepaired somatic cells for disease modeling or drug screening.

The treatment of many diseases is difficult because of the lack of information about the mechanisms that play a role in the disease progression. For this reason, diseases need to be modeled so that treatment could be developed aiming the main cause of the disease. There are a large number of disease testing models which have developed during previous era. Some of them are capable of mimicking human cellular microenvironment and metabolism to some extent. Many animal models such as rat, mice, dogs monkey, dog, and primates have been used for disease modeling. However, the use of animals as disease models is limited due to existing variability in the genetic make-up of them that is highly responsible for the biological functions and hence differences are exhibited while compared with human individuals. Secondly, the problem further gets complicated when the individuals are of two different species. Different species have different genetic makeup and hence different proteins. And thus, none of the animal model is able to fully mimick the human cell microenvironment. So, a different approach which can provide the same environment as in human cells is required and iPSCs pose to be a good alternative with some advantages as well. In case of iPSCs, there is no need of proliferation again and again, and, their derivatives are functional *in-vitro* as well as *in-vivo* after transplantation. Induced pluripotent stem cells are widely used in therapeutics for disease modeling, regenerative medicine, and drug discovery (Figure 4).

### Disease modeling

The use of iPSCs for disease modeling is based on the fact that these cells are capable of self renewing and that these cells can differentiate into all types of cells of the human body which can be utilized for the preparation of different disease models to study those diseases. Moreover, a patient specific iPSC could be of enormous use as far as development of specific therapeutics regimen/drug is concerned. By combining 3D culture with extracellular matrix proteins, *in-vivo* microenvironment can be mimicked. Lee et al. used iPSCs for the modeling of pathogenesis in Familial Dysautonomia (Lee et al., 2009). Since then, there have been many cases in which iPSCs have helped out in studying various mechanisms that play role in different diseases, a few have been described below.

Moad et al. used human prostate and urinary tract cells for the formation of iPSCs and further for studying the mechanisms that regulate the differentiation of prostate and urinary tract cells. With their study, they reported the first successful reprogramming of bladder, prostate and ureter stromal fibroblasts into a pluripotent state and concluded that iPSCs generated from prostate and urinary tract had better efficiency of differentiation to cells of prostate and urinary tract as compared to iPSCs derived from skin fibroblasts which showed that organ of origin plays an important role in terms of efficiency of differentiation (Moad et al., 2013).

Various types of diseases which are caused by some deficiency have been studied by using iPSCs. Park et al. used iPSCs from patients of various diseases like *Adenosine Deaminase Deficiency-related Severe Combined ImmunoDeficiency (ADA-SCID), Shwachman-Bodian-Diamond syndrome (SBDS), Gaucher disease (GD) type III* for the study of disease models and drug discovery. They used dermal fibroblasts or bone-marrow derived mesenchymal stem cells for the generation of human iPSCs by the transduction of all four or three (excluding c-Myc) transcription factors. It was found from their study that ADA-SCID, SBDS, and Gaucher's disease type III are inherited in a classical Mendelian Inheritance manner like congenital disorders which are autosomal recessive. These diseases were shown to be caused by point mutations in those genes which were vital for normal hematopoiesis and immunological function. They also reported the point mutations in ADA-SCID, SBDS, and GD type III. They also studied *Duchenne (DMD) and Becker muscular dystrophy (BMD), Parkinson disease (PD), Huntington disease (HD), juvenile-onset, type 1 diabetes mellitus (JDM), Down syndrome (DS) /trisomy 21 and the carrier state of Lesch-Nyhan syndrome* (Park et al., 2008b).

There are many syndromes which are caused by the existence of one or more extra copies of a chromosome. Downs syndrome is one such example. Briggs et al. used iPSCs for the identification of molecular networks that drive the different aspects related to pathogenesis in Down's Syndrome. iPSCs in combination with microarray and RNA sequencing technology, can be used to generate phenotype-genotype maps of complex diseases by linking various defects with phenotypes, like in Down's Syndrome using Chromosome engineering of Down Syndrome-iPSCs (Briggs et al., 2013).

Neurogenerative diseases have also been studied using iPSCs as disease models. Parkinson's Disease (PD) is a very common neurodegenerative disease, in which, dopaminergic neurons of substantia nigra (a structure in mid brain) get lost and formation of Lewy's bodies (inclusions in the cytoplasm of neurons all over the body) occurs. Treatment of this disease had not been possible due to the reason that by the time, PD gets clinically manifested, the neurons have already lost, which makes it very difficult to be able to study the underlying mechanisms of PD so as to develop a treatment of it. In such a situation, iPSCs can be used and experiments have also been carried out in this aspect. Nguyen et al. studied G2019S mutation in LRRK2 (Leucine Rich Repeat Kinase2) gene. This mutation has been reported in cases of sporadic and familial PD. Fibroblasts from a 60-year old female homologous for G2019S mutation were used as a source for the generation of iPSCs. Retrovirus was used for the transduction of Oct4, Sox2, and Klf4 into the fibroblasts, which after 2 months of transfection developed 3 colonies of human ESC, which were analyzed and found out to be similar to hESCs and wild type iPSCs. For differentiation of G2019S iPSCs to dopaminergic neurons, a combination of mib-brain selective patterning factors, sonic hedgehog (SHH) (induces the differentiation of dopaminergic neurons in substantia nigra), and Fibroblast growth factor 8 beta (FGF8b) [induces the development of cerebellum by the activation of Ras-extracellular signal- regulated kinase (ERK) pathway] and small molecules was used. The neurons generated by this method were functionally active and were found to be able to form active neural networks. The findings suggested LRRK2 to be having an important role in survival of neurons and disruption of which can cause PD. (Hurtado-Lorenzo et al., 2004; Sato et al., 2004; Nguyen et al., 2011). Similarly, a different group has also worked for the generation of iPSCs in PD. Devine et al. developed iPSCs from fibroblasts taken from a PD affected person possessing triplication of Synuclein gene by the transduction of four basic transcription factors. These iPSCs were then directed to differentiate into dopaminergic neurons *in vitro* for the study of PD (Devine et al., 2011).

These findings from modeling different diseases (mutations which are causing them, the pathological phenotypes caused by defects in various networks, genes playing an important role) help out in knowing the molecular mechanisms underlying the disease better, which ultimately carries the work forward to knowing the disease better for the development of a treatment. This is evident from burgeoning reports on use of IPSc for the the purpose of disease modeling (Table 4). However, the technique suffers from various inherited risks and limitations as discussed ahead.

**Table 4:** **List of diseases where iPSCs have been used for gene therapy and disease modeling**.

**Disease/syndrome**	**Cause(s)**	**Features**	**Cells used for iPSC generation**	**System used**	**References**
Parkinson's Disease (PD)	Familial forms caused by α-synuclein, ubiquitin carboxy terminal hydroxylase L1, parkin, DJ-1, putative serine threonine kinase 1 and leucine rich repeat kinase 2	Loss in nigrostriated dopaminergic neurons in substantia nigra; presence of Lewy bodies	Dermal fibroblasts of patient with idiopathic PD	Lentiviral	Soldner et al., 2009
Huntington disease (HD)	CAG repeats (36 or more) in the first exon of *htt* gene gets expanded abnormally	Degeneration in striatum and cerebral cortex	Fibroblasts	Lentiviral	Park et al., 2008b; Kaye and Finkbeiner, 2013
ALS or Lou Gehrig's disease	Autosomal dominant mutation in superoxide demutase (SOD1)	Death of motor neurons of the motor cortex, brain stem and spinal cord	Fibroblasts	Lentiviral	Rosen et al., 1993; Chestkov et al., 2014
Friedreich's ataxia (FRDA)	GAA trinucleotide repeat in the first exon of the frataxin gene gets expanded	Accumulation of mitochondrial iron, specific enzymes in mitochondria become defective, sensitivity to oxidative stress increases, cell death mediated by free radicals	Fibroblasts	Lentiviral	Campuzano et al., 1996; Ku et al., 2010
Lesch-Nhyan syndrome (carrier state)	Deficiency of hypoxanthine guanine phospho ribosyl transferase (HPRT)	Over-production of uric acid, low or medium level of mental retardation, megaloblastic anemia is frequent	Dermal fibroblasts	Lentiviral	Park et al., 2008b
Shwachman-Bodian-Diamond syndrome (SBDS)	Mutations in the Shwachman-Bodian-Diamond syndrome (SBDS) gene	Exocrine pancreatic insufficiency, predisposition to leukemia, hematopoietic dysfunction	Fibroblasts	Lentiviral	Tulpule et al., 2013
Gaucher's type III	Deficiency of acid hydrolase, β-glucocerebrosidase, or glucosylceramidase	Myoclonal epilepsy, nerve deafness	Fibroblasts	Lentiviral	Park et al., 2008b
Becker type muscular dystrophy (BMD)	Mutation in dystrophin gene	Loss of walking ability, but progression slower than DMD	Fibroblasts	Lentiviral	Park et al., 2008b
Downs syndrome/trisomy 21	Trisomy of chromosome 21	Cardiac and cognitive defects, premature Alzheimers disease and aging, dysmorphic facial features	Fibroblasts	Lentiviral	Park et al., 2008b; Briggs et al., 2013
Familial dysautonomia (FD) or Riley-Day syndrome	Autosomal recessive disorder caused by a single mutation in exon 20 in I-K-B kinase complex associated protein (IKBKAP) gene	Dysfunction of small fiber sensory neurons	Fibroblasts	Lentiviral	Lee and Studer, 2011
Childhood cerebral adreno leuko dystrophy (CCALD)	Mutation in *ABCD1* gene	Adrenal cortex, nervous system and testes get affected, leading to rapid cerebral demyelination and adrenocortical atrophy.	Skin fibroblasts	Retroviral	Wang et al., 2012
Rett's syndrome	Classic form caused by loss-of-function mutation in Methyl-CpG-binding protein 2 (MECP2) gene on the X - chromosome, variants caused by mutations in FOXG1 or CDKL1 on chromosome 14 and X-chromosome, respectively	Neurocognitive regression and autistic behavior	Fibroblasts	Retroviral	Amenduni et al., 2011; Farra et al., 2012
Duchenne type muscular dystrophy (DMD)	Biochemical and genetic defects in Dystrophin-glycoprotein complex	Loss of walking ability	Tail tip fibroblasts (mouse)	Retroviral	Gangopadhyay et al., 1992; Filareto et al., 2013
Generation of human prostate and urinary tract cells	NA	NA	Human prostate and urinary tract cells	Lentiviral	Moad et al., 2013
Adenosine deaminase deficiency-related severe combined immunodeficiency (ADA-SCID)	Defects in Adenosine deaminase (AD) gene	Impaired development and functioning of T, B, and NK cells; complete absence of humoral and cellular immunity; recurrence of infections.	Bone Marrow derived mesenchymal cells	Lentiviral	Park et al., 2008b; Sauer et al., 2012
Type 1 diabetes mellitus (DM)	Progressive β-cell destruction	Long term micro and macro-vascular complications.	Fibroblasts	Lentiviral	Park et al., 2008b; Soejitno and Prayudi, 2011
Hemophilia A	Deficiency of factor VIII	Decreased protein production, inefficient clotting of blood	Fibroblasts	Retroviral	Xu et al., 2009
Glycogen storage disease 1a	Mutation in Glucose-6-Phosphate gene	Increased accumulation of lipids and glycogen	Dermal fibroblasts	Retroviral	Lei et al., 1995; Rashid et al., 2010
Familial hypercholestrolaemia	Mutation in low density lipoprotein receptor (LDLR) gene	Deficiency of LDL-receptor mediated uptake of cholesterol	Dermal fibroblasts	Retroviral	Rashid et al., 2010
Spinal muscular atrophy	Mutation in survival of motor neuron 1 (SMN1) gene	Paralysis, muscle weakness and often death	Fibroblasts	Lentiviral	Ebert et al., 2009
Hutchinson-Gilford progeria syndrome	Point mutations in lamin A	Premature atherosclerosis, vascular smooth muscles gets degraded	Fibroblasts	Retroviral	Liu et al., 2011a
Alzheimer disease	Duplication of amyloid β precursor protein (APP)	Presence of neurofibrillary tangles and amyloid plaques in the brain	Fibroblasts	Retroviral	Israel et al., 2012
LEOPARD syndrome	Mutation in protein tyrosine phosphatase non-receptor type 11 (PTPN11) gene	Cardiac abnormalities, ocular hypertelorism, and growth retardation.	Fibroblasts	Retroviral	Legius et al., 2002; Carvajal-Vergara et al., 2010.
Timothy's syndrome	CACNA1C	Webbed fingers and toes, autism, immune deficiency	Fibroblasts	Retroviral	Yazawa et al., 2011
Dyskeratosis congentia	Mutation in dyskerin (DKC1) gene	Increased failure of bone marrow, pulmonary fibrosis and cancer, oral leykoplakia, abnormal skin pigmentation and nail dystrophy	Fibroblasts	Retroviral	Batista et al., 2011
α1-antitrypsin deficiency	Mutation in α1-antitrypsin (A1AT) gene	misfolded α1-antitrypsin gets aggregated in the endoplasmic reticulum	Dermal fibroblasts	Retroviral	Rashid et al., 2010

### Regenerative medicine

In regenerative medicine the injured or degenerated tissues are repaired by the generation of those tissues with the help of iPSCs in labs and then transplanting them to the site of injury or degeneration. Important issues associated with gene therapy are availability of tissue or organ and immunorejection. It is quite often that patients die because of the non-availability of donors which is a result of the fact that the population is non-aware of the scarcity of donors and the ever inceasing need of organs due to accidents and degenerative diseases. Also, a patient can only be transplated with the cells, tissues, or organs from the person who does not have any disease and whose physiological profile matches with the patient. Keeping these risks in mind, various tests are conducted before transplating tissues or organs into the patient's body. The use of iPSCs offers a good approach for these treatments as the cells that will be transplanted to patient's body will be differentiated from the repaired iPSCs generated from the somatic cells from patient's own body. iPSCs have been used in treating a number of injuries and degenerative diseases. A comprehensive detail of all those would be beyond the scope of this article, however, major arguments are discussed below. Different injuries as a result of human activities, accidents or natural calamities can also be treated by the gene therapy utilizing iPSCs. The various conditions that can be treated are Hematopoietic disorders, Musculoskeletal injury, Spinal cord injury, liver damage by the generation of specific cells with the help of iPSCs (Liu et al., 2011b; Nori et al., 2011; Tan et al., 2012; Suzuki et al., 2013).

Other than accidental injuries, diseases can also be treated with the help of iPSCs. Kazuki et al. corrected the genetic deficiency in iPSCs from a human Duchhene Muscular Dystrophy (DMD) patient. They used Human Artificial Chromosome (HAC) for the expression of complete sequence of Dystrophin (DYS). They used fibroblasts from the DMD patient for the generation of iPSCs. HAC was used for the correction in the deletion or mutation in the Dystrophin gene present in iPSCs by transfer of DYS-HAC (complete genomic sequence of dystrophin in HAC) using Microcell-mediated chromosome transfer (MMCT) (Kazuki et al., 2009). MMCT is a technique in which micro cells containing few chromosomes are fused with whole cells (Doherty and Fisher, 2003).

iPSCS can also be used in cases of hepatocytes where loss of function of hepatocytes in culture and limited organ availability act as obstacles when fetal or adult progenitors are used for the development of hepatocytes. This lead to making use of iPScs for the generation of hepatocytes for various liver problems.

Recently, there are accumulating data on the use of iPSC for *ex-vivo* blood expansion of various blood components. They can be used for the generation of Red Blood Cells (RBCs) which could be utilized for the generation of blood that is required over the globe for the purpose of treatment of various damages or diseases. There are various techniques by which we can use ESCs/iPSCs for the production of RBCs (Lim et al., 2013).

iPSCs can also be used for the generation of various cells which can help in the repairmen of many tissues, for example, cardiovascular cells for the repairment of heart valves, vessels and ischemic tissues, but are limitations like safe delivery, post treatment adverse effects and standardization of protocols to generate large amounts of pure good quality cells. These obstacles once overcome, offer great opportunities for the applications of iPSCs for generating cardiovascular cells and studying corresponding diseases (Laflamme et al., 2007; Cao et al., 2008; Levenberg et al., 2010).

Various degenerative diseases in which cells death causes many symptoms have been treated by gene therapy using iPSCs. One such case is Retinitis pigmentosa (RP) where eye's retina degeneration causes impaired vision. For the treatment of RP, iPSCs were generated from the patient suffering with the disease which were then showed to differentiate into rod photoreceptor cells (Yoshida et al., 2014). The differentiation of iPSCs to Retinal pigmented epithelium is proved to be beneficial for patients of Retinal Pigmentosa and Age-related Macular Degeneration (AMD) (Li et al., 2012).

Gene therapy using iPSCs has also been used to treat various primary immunodeficiencies like *Chronic Granulomatous disorder (CGD)*, an autosomal recessive or X-linked disorder which affects the function of neutrophils, and *Wiskott–Aldrich syndrome (WAS*), X-linked deficiency caused by mutation in WAS gene encoding WAS protein (WASp) which is an actin cytoskeletol regulator in hematopoietic lineage (Klein et al., 2003; Jiang et al., 2012).

The technology of genome editing or genome engineering, which includes the use of different genome editing technologies like Zinc Finger Nucleases (ZFNs), Transcription Activators like effector nucleases (TALENs), CRISPR/Cas systems, Meganucleases, Adeno-associated viruses, and adenoviruses, offers an opportunity for the efficient introduction of various insertions or deletions that range from single nucleotides to insertion/deletion of whole genes which has a capability of taking disease modeling to new heights (Suzuki et al., 2008; Grizot et al., 2009; Hockemeyer et al., 2009, 2011; Khan et al., 2010). Choi et al. reported the gene correction in alpha 1 anti trypsin (AAT) deficiency by the use of TALENs. A pair of TALEN expression vectors was constructed. This construct recognized the sequences flanking the Z mutation of the AAT gene. After co-nucleofection of targeting vector and TALEN expression vector was carried out, PCR and Southern blot was done. The results showed a highly efficient and simultaneous targeting of both alleles (Choi et al., 2013).

Once the specific cells are formed they can be transplanted to the specific site, in case of degenerative diseases, for the cure of the disease. And, in case where diseased cells which possess some mutation, the mutation is first corrected to form normal iPSCs and then these iPSCs are differentiated into specific cell types by providing the specific conditions required for the development of those cells. These repaired cells can then be transplanted into the body of the organism from which the cells for the generation of iPSCs were isolated.

### Drug discovery and cytotoxicity studies

Another important role offered by the use of iPSCs based methodology is their complementarity to drug discovery or prediction of toxicity via animal models. Animals or *in-vitro* animal derived cells are used as testing systems butare limited by their inability to to replicate the “exact” human physiological conditions and related phenotypic attributions. Sometimes, the benefits demonstrated in the animal models do not come out to be beneficial in humans, for example, the SOD (superoxidase dimutase) gene associated with ALS (Amyotrophic Lateral Sclerosis) allowed the identification of Vitamin E and Creatine to be relievers of the diseased phenotype which failed to cause any improvements in humans (Desnuelle et al., 2001; Groeneveld et al., 2003; Shefner et al., 2004). And also, animal models are not good testing models for drug toxicity as a chemical may be toxic to an animal but may not be toxic to a different animal. For carcinogenic agents as well, different agents pose different levels of carcinogenicity in different animals, for example, formaldehyde is more carcinogenic to rats as compared to mice (Kerns et al., 1983). Finally, a newly discovered drug or therapy must be tested on human cells or human test models itself. These reasons make it more important to be able to use the systems closer to humans. Moreover, these studies need to be done in a system where the results could be directly extrapolated to humans. These studies include steps such as prediction/identification of a potential drug molecule followed by its synthesis, generation of iPSCs, their differentiation to specific somatic cells, and testing for toxic or non-toxic effects of the synthesized drug on the somatic cells. For toxicity studies, iPSCs from normal and diseased cells are used to generate neurons, hepatocytes, cardiomyocytes etc. Toxicity and potential side-effects are often most common cause to rule out most of the therapeutic molecules.

There is a long pipeline between the time when a hit is identified and the time when it reaches the market. Before using drugs on humans, their toxic effects must be properly evaluated for a safe administration of those drugs which is very costly. Only 10% of the drugs that enter clinical trials are able to reach market approval stage. The cost of developing a drug is increasing with the estimated cost of whole process being US $1.2–1.7 billion per drug compound (Kaitin, 2008; Sollano et al., 2008; Gunaseeli et al., 2010). The development of 30% of the medicines was abandoned because of lack of efficacy and 30% due to concerns associated with safety (cardiotoxicity, hepatotoxicity) (Laustriat et al., 2010). The blocking of human ether-a-go-go related gene 1 (*hERG1*) channel by drug is associated with longer duration of ventricular repolarization which causes long QT syndrome. The response of iPSC-derived cardiomyocytes have been reported in response of cardiac and non-cardiac drugs in terms of electrophysiological capacity (Tanaka et al., 2009; Yokoo et al., 2009; Asai et al., 2010; Yoshida and Yamanaka, 2010). The generation of functional hepatocytes using iPSCs has also been reported (Sullivan et al., 2010). Due to lack in early detection of drug toxicity in human tissues, the process becomes more costly and extensively time consuming. Development of toxicity models that predict more accurately before clinical trials may help to reduce costs by demonstrating cardiotoxicity or hepatotoxicity caused by the drugs much before they reach clinical trials; reducing the time taken for clinical trials of the drugs which will fail due to cytotoxicity in the later stages of trials. iPSCs have been explored by many research groups in order to identify compounds which are toxic or pose side-effects. The use of iPSCs offer better alternative to conventional tests of toxicology and drug research and offer better chemical safety assessment as they provide a more similar environment to human physiological conditions than is provided by conventional testing systems which make use of animals. For example, iPSCs have been used to establish test systems for cytotoxicity–cardiotoxicity, hepatotoxicity, and embryotoxicity testing (Kimmel and Young, 1983; Seiler et al., 2004).

iPSCs have the potential of providing native cells for splicing and post translational modifications that does not come with the small molecule screening systems using purified protein or recombinant cell lines used for the over-expression of the protein of interest which has aided in the process of drug screening. The use of iPSCs in drug screening has also been reported. Stem Cells enable the identification of molecules therapeutically useful and able to modulate the behavior of tumorigenic and normal stem cells. But as the use of ESC is limited because of the concerned ethical issues, iPSCs (normal and tumorigenic) can be used as an alternative.

McNeisch et al. used neurons that were expressing a-amino- 3-hydroxyl-5-methyl-4-isoxazoleproprionate (AMPA) subtype glutamate receptors using mouse iPSCs and used them for the identification of small molecule AMPA potentiators (McNeish et al., 2010). Choi et al. used hepatic cells derived from iPSCs generated from patients with alpha-1 anti-trypsin deficiency for the screening of 3131 compounds of Johns Hopkins Drug Library. After immunofluorescence, total AAT fluorescence intensity was measured for each well. Out of 262 compounds which inhibited the accumulation of AAT by more than 50%, 43 compounds after literature survey were found to be FDA approved, clinically used internationally, and exerting no major side effect. These compounds were further tested by using four different lines of AAT- deficiency iPSCs and final 5 hits, which gave consistent results in all the four iPSC lines were selected (Choi et al., 2013). iPSCs also help in studying ADME (Absorption, Distribution, Metabolism, Excretion) properties of drugs which has highly speeded up drug discovery and development (Bahadduri et al., 2010).

## Advantages of iPSC technology

iPSCs offer various advantages as compared with gene therapy by the use of nuclear transfer or Embryonic Cell mediated gene therapy (Kazuki et al., 2009).

### The ethical issues are elimited by the use of iPSCs

The use of ESCs in research is laden with ethical issues regarding personhood, justice toward humankind and human dignity that are associated with the use of human life in its earliest form, the embryo. Additional concerns that surround the use of ESCs are informed consent, improper incentives, and health and safety concerns of the women donating eggs for the generation of embryos by *in-vitro* fertilization. iPSCs have solved the controversy over the destruction of embryos associated with the use of ESCs in research.

### Reduced chances of immunorejection

iPSCs are generated from the somatic cells of one's own body and hence there is no risk of immunorejection of these autologous cells. Guha et al. differentiated mouse iPSCs into embryoid bodies and different tissue specific cells. After the transplantation of these iPSC derived EBs or tissue specific cells, no evidence of increased Tcell proliferation or an antigen- specific secondary immune response was found (Guha et al., 2013).

### Throughput screening for predicting toxicity/therapeutic responses of newly developed drugs

The concept of using iPSCs to predict toxicology and therapeutic responses of drugs in based on the property of iPSCs to continuously self renew which make it possible to generate libraries and their ability to give rise to all types of body cells make them suitable to be used for prediction of toxicity and possible side effects of newly developed drugs in different body cells (Wobus and Loser, 2011).

### Lowering the overall cost and risk of clinical trials

For every new drug to reach market, around 5000–10,000 compounds need to be tested during preclinical trials. Therefore, any strategy which standardizes the prediction of toxicity would impact the cost. Many drugs fail after Phase III, for example BMS-094 (hepatitis C drug) failed after death of a patient. The cost used for the clinical trials could be reduced by using iPSCs to provide the toxicity details of the drug by different cytotoxicity assays. Much of the cost for preclinical testing is due to the requirement of animal models for estimation of bioavailability of new drug. As animal models do not mimic the microenvironment of the human cells fully, the use of iPSCs for these tests may cut the cost associated with providing animal models which will ultimately decrease the overall costs of the clinical trials.

### Development of a personalized approach for administration of drugs

As, iPSCs are derived from individual patients, these offer scientists an opportunity for modeling diseases on a patient-by-patient basis. This enables screening the genomic differences between individuals that may help in the progression of disease, and the screening of pharmacological agents to find the ideal one for each individual (Chun et al., 2011).

### Gene targeting and correction technologies (gene therapy)

Reprogramming of somatic cells from any genetic background to iPSCs has allowed the generation of cell lines possessing disease-causing mutations. The ability of modifying the specific sites in a genome for altering the genes of interest becomes highly important here. To realize the full potential of iPScs, methods for efficient genetic modifications are required.

### For disease modeling, the phenotypes need to be consistent every time, which is a possibility in case of iPSCs

Many research groups have modeled various diseases using iPSCs and the cell lines developed were found to be consistent phenotypically. Fong et al. developed Tauopathy derived iPSCs carrying a TAU-A152T mutation. The phenotypes observed in the cells from iPSCs were consistent with those in patients with the mutation (Fong et al., 2013).

## Limitations and/or risks associated with the use of iPSCs IC clinics

There are some limitations/disadvantages associated with iPSCs as well. Generation of iPSCs make use of retroviral or lentiviral systems, so, it needs to be concerned if the viral systems get incorporated with the host genome. The genetic material inserted via retroviral vectors may randomly integrate into the genome of the host which can cause genetic aberration and teratoma formation (Howe et al., 2008). The transcription of the transgenes inserted via vector systems may resume in the cells that have been formed by the differentiation of iPSCs (Okita et al., 2007). The assessment also becomes complex as it depends on number of variables, like small molecules for the enhancement of iPSCs production are used or not, whether the factors used for reprogramming are contained in single vectors or multiple vectors, and which source has been used for the generation of iPSCs, contribute to the differences in the epigenome, genotype, and phenotype of the iPSCs produced which make it difficult to determine whether the problems occurred due to the technology or the reprogramming method(s) (Howe et al., 2008; Li et al., 2011). It is possible to use iPSCs for modeling of the diseases which have a phenotype and are monogenic but in case of complex diseases the role of genetic and epigenetic factors is uncertain which makes modeling of disease a tough job. It is not possible for iPSCs to model every disease successfully, hence, it should be noted if the iPSCs derivatives are getting properly matured to the cells of interest or not in order to know if they are suitable to be used for the purpose of our interest or not. The altered expressions of the basal reprogramming factors has also been reported to cause diseases. The overexpression of Oct4 may lead to epithelial cell dysplasia (Hochedlinger et al., 2005). The aberrant expression of Sox2 has been reported to cause mucinous colon carcinoma (Park et al., 2008c). Klf4 has role in the formation of breast tumors (Ghaleb et al., 2005). c-Myc plays an important role in the formation of around 70% of human cancers (Kuttler and Mai, 2006). For this reason, retroviral introduction of c-Myc is also advised to be avoided for use in clinical applications (Okita et al., 2007). Zhang et al. reported that out of 593 genes that express in iPSCs, 209 genes were found to express in cancer tissues and tumor cells. Five oncogenes were found to overexpress in iPSCs whereas the oncogene RAB25 was found to express in cells derived from iPSCs (Zhang et al., 2012). RAB25 (member of RAS protein family) plays an important role in survival of cancer cells (Cheng et al., 2013). The different pros and cons of the use of iPSCs in different applications has been mentioned in Table 5.

**Table 5:** **The pros and cons associated with the use of iPSCs**.

	**Pros**	**Cons**
Due to characteristics of iPSCs	Eliminates ethical issues	Premature aging
	Reduced chances of immunorejection (Guha et al., 2013)	High rate of apoptosis
	Reduced risks of clinical trials	Low level DNA damage repair (Zhang et al., 2012)
	Consistent phenotypes for disease modeling (Fong et al., 2013)	Sensitive to ionizing radiation (Zhang et al., 2013)
	Differentiation to any cell type	Low rate of reprogramming
Due to technology of development	Continuous cell supply	Insertional mutagenesis (Okita et al., 2007; Howe et al., 2008)
	Possible preservation	Tumourogenesis (Okita et al., 2007).
	Availability and accessability of source cells	Chances of development of diseases due to factors used (Ghaleb et al., 2005; Hochedlinger et al., 2005; Kuttler and Mai, 2006; Park et al., 2008c)
	Personalization of treatment (Chun et al., 2011)	Suboptimal standardization (Pappas and Yang, 2008)
Applications	High-throughput screening of drugs and toxicity prediction (Wobus and Loser, 2011; Choi et al., 2013)	Complex assessment
	Reduced cost	Complex diseases become difficult to be modeled
	Gene correction therapies add to the benefits from iPSCs (Choi et al., 2013)	Immature cells cause problems during cell line development

## Quality control testing of iPSCs

There are many things which need to be considered before using iPSCs clinically for regenerative medicine. The manufacture of clinical grade iPSCs demand quality control at different levels of the manufacturing process. Firstly, it is important to decide whether the therapy would be autologous or allogeneic. Autologous therapy should be such that it is able to manage the differences in the patient in terms of age, health and gender which could affect the quality, consistency and safety of the final product. It requires long term study of the increased risk of tumor formation as the patient would not be expected to have any immune response. The alternative, allogeneic therapy offer well-characterized cell banks for therapeutics to be established. Donors need to meet strict eligibility requirements and the donor must be aware of the use and testing of his sample and that the collection of sample would be carried out in an ethical way. After the collection and culture of the source cell, a suitable reprogramming method is to be decided. The use of retroviral vectors has risks of insertional mutagenesis and hence their use is not ideal for clinical applications. Non-viral methods address this question and the method is chosen according to pros and cons associated. Once the iPSCs have generated, proper characterization is vitally required. For the verification that the iPSCs generated are really pluripotent, the expression of different pluripotency markers is assessed. It must be checked if the iPSCs generated can differentiate to all the three germ layers. To further check the ability of the iPSCs to differentiate, the efficiency of generation of the cells which are required for the therapy is determined. The cells generated must be tested to be functional, so the physiological properties of cells generated from iPSCs were compared with that of the cells generated from ESCs. The final product has variability which depends on the quality of starting material used as cell source, difference in other raw materials used for cell culture, and the protocol followed. Identifying and controlling these is important to standardize any manufacturing process to demonstrate reproducibility and consistent production meeting the specifications. Research based methods that have manual nature may not prove to be ideal for the large production of cells. Quality control testing for final product and preparation procedures must also be designed. For administration of fresh cells, there is need of a rapid method for detection of contamination. Plans must be there in case if the sample fails the sterility test, if the patient has already been administered with the product. Once the quality testing has been done, it should be ensured that the final cell therapeutic is meeting all the required specifications. For allogeneic therapies, a cell bank must be established which would contain the starting cell material and it should be tested to check the consistency of that material. Establishment of a large cell bank is not important for autologous therapeutics; a small bank would serve the purpose of identification and minimization of variability that may result from initial reprogramming. The colonies which meet the specifications of karyotype, marker expression, mycoplasma/bacterial/fungal contaminate and growth characteristics, are expanded to build a bank. Additional knowledge about the post release handling of the cell product is also required. It is important to ensure that the final cell product is representative of therapeutic that will be subjected to human clinical trials. Preclinical animal studies should be able to address the safety of the cell product which includes the affects of the contaminants present in the cell population from donor, chances of teratoma formation and generation of karyotypically unstable cells. It should be ensured that the changes in process associated with change from animal studies to production for clinical use would not cause any significant problems.

## Future perspectives

Since the emergence of the field of iPSCs, it has highly developed and successfully expanded to different fields of regenerative medicine. Their application in clinical research will surely benefit the patients in future. At present there are few, but important applications of iPSCs but, if this field keeps on growing at the present pace, it wouldn't take long to expand the applications of iPSCs to more biological fields to aid research and treatments. iPSCs are important in disease modeling as they do not have ethical issues concerned as in case of ESCs and provide with the physiological conditions more close to humans which is not an advantage with the animal disease models. iPSCs also have a possibility of giving consistent phenotypes every time, which is an important issue for disease modeling. More and more types of diseased cells are being tried to be modeled, but, it still has many limitations that are encountered while working with iPSCs, which need to be overcome. Also, it is not possible to be able to model every disease, so, further exploration of modeling diseases with iPSCs is required. As iPSCs eliminate the chances of immunorejection, they hold great importance in Gene therapy. But the generation of iPSCS itself has many issues associated it like incorporation of vectors into host genomes, and the dependence on the various factors like usage of small molecules, single or multiple vectors, source of cells, which make the generation of iPSCs a risky task and hence, more about iPSCs need to be explored to make it easier to generate them and to be able to apply them to various other important and attractive areas of medical sciences for their proper utilization to achieve the advantages that use of iPSCs can have. And, different advances in the field, including the solution of many limitations mentioned above would prove to be beneficial for increasing therapeutic potential. The present situation of the applications of iPSCs deals with the laboratory scale production and testing assay. A lot has to be done to realize the applications of iPSCs at a higher level, for the use in hospitals, to be beneficial for the patients.

## Conclusion

The iPSC therapy is in infancy but a constant progress in reprogramming technologies has occurred; new and improved methods are gathering. As iPSCs can differentiate to different cell types, we can generate the required cells for the study or treatment of numerous diseases and determination of drug toxicity. The therapy using HSCs is being used since a long time. The change from allogeneic to autologous iPSCs has been a major breakthrough. Autologous therapy also offers the advantage of personalized medicine.

### Conflict of interest statement

The authors declare that the research was conducted in the absence of any commercial or financial relationships that could be construed as a potential conflict of interest.
